# Essence of postmortem computed tomography for in-hospital deaths: what clinical radiologists should know

**DOI:** 10.1007/s11604-023-01443-w

**Published:** 2023-05-17

**Authors:** Masanori Ishida, Wataru Gonoi, Hiroyuki Abe, Tetsuo Ushiku, Osamu Abe

**Affiliations:** 1 Department of Radiology, Graduate School of Medicine, The University of Tokyo, 7-3-1 Hongo, Bunkyo-Ku, Tokyo, 113-8655 Japan; 2 Department of Pathology, Graduate School of Medicine, The University of Tokyo, 7-3-1 Hongo, Bunkyo-Ku, Tokyo, 113-8655 Japan

**Keywords:** Postmortem computed tomography, Postmortem imaging, In-hospital deaths, Cause of death, Post-cardiopulmonary resuscitation change

## Abstract

Postmortem computed tomography (CT) is an essential tool for investigating the causes of death. Postmortem CT has characteristic imaging findings and should not be interpreted in the same manner as clinical antemortem images. In analyzing the cause of death in in-hospital death cases using postmortem images, it is crucial to understand early postmortem and post-resuscitation changes. In addition, it is essential to understand the limitations of diagnosing the cause of death or significant pathology relating to death on non-contrast-enhanced postmortem CT. In Japan, there has also been social demand to establish a system for postmortem imaging at the time of death. To facilitate such a system, clinical radiologists should be prepared to interpret postmortem images and assess the cause of death. This review article provides comprehensive information regarding unenhanced postmortem CT for in-hospital death cases in daily clinical practice in Japan.

## Introduction

Postmortem imaging is the examination of corpses to investigate the cause of death or pathology related to death, and enables an observation of the inner body which cannot be obtained from the surface. In Japan, prior to the emergence of the concept of postmortem imaging, postmortem computed tomography (PMCT) of in-hospital deaths was reportedly performed in emergency rooms as there was a low rate of autopsy and a wide availability of CT scanners [1]. Eventually, in Japan, the concept of “Autopsy imaging” was proposed in 2000 [2]. Nowadays, it is locally abbreviated as “Ai”, and PMCT has become an essential tool for death investigation [3]. According to a 2021 report of the Japanese Ministry, 34.5% of unusual death cases (17,0174 out of 58,689 cases) underwent PMCT in 2018 [4].

The annual number of deaths in Japan began to exceed the number of births in 2005 [5]. If this trend continues, the number of deaths outside medical institutions, such as homes, is expected to increase [6]. Therefore, there is an increasing importance and need for cause-of-death investigations, which serves to improve public health and averts preventable deaths. In 2012, the law concerning the “Act to Accelerate Investigation into the Cause of Death and Identification” was enacted in Japan [7]. Further, in 2015, the “Medical Accident Investigation System” was established to prevent repeated medical accidents [8]. The Japanese system for investigating causes of death is being strengthened, and the use of imaging modalities at the time of death is expected to improve the accuracy of death-cause diagnosis. In this context, diagnostic guidelines for postmortem imaging in Japan were established and translated into English and Italian to standardize the interpretation of postmortem imaging [9].

This narrative review article describes essential knowledge required of clinical radiologists when interpreting postmortem non-contrast-enhanced CT of in-hospital death cases.

## Practical operation of postmortem CT in in-hospital death cases

In most cases of postmortem imaging for in-hospital deaths in Japan, the time from death to postmortem CT scanning is several hours to 1 day, and the patient is kept in a supine position in a mortuary until postmortem CT scanning [10–12]. Postmortem CT of the deceased does not produce respiratory or motion artifacts. Dose reduction techniques are not necessary to reduce radiation exposure. Therefore, there is no problem with imaging under conditions that provide the best image quality, but an increased dose does not always improve image quality [13]. It is essential to include coverage from head to pelvis during the scanning, and the scanning range may be divided between the head and trunk. If the lower extremities are presumed to be less involved in the cause of death, they could be omitted [13]. During scanning, the standard position for the body is supine, with both upper extremities in the drooping position. Artifacts on the trunk caused by drooping of the upper extremities may degrade image quality [14]. In cases where medical malpractice is suspected, it is crucial to preserve the circumstances at the time of death by scanning CT images with medical devices, such as catheters and tubes, left in place [15].

## CT findings of normal postmortem change

CT imaging can reveal the various changes that occur in the body after death, known as postmortem changes. These changes are classified into early (occurring within 24 h of death) and late postmortem changes [16]. Examples of early postmortem changes include pallor mortis (the earliest change which reflects the external skin color change), algor mortis (the progressive decrease in body temperature), livor mortis as known postmortem hypostasis (the non-circulating blood and other fluids settle with gravity toward the dependent parts of the body), and rigor mortis (stiffening of muscle). However, some changes, such as pallor mortis and algor mortis, cannot be visualized on CT scans [17], although low temperature induces density changes in tissue and associated CT value changes. Meanwhile, findings reflecting the cessation of circulation are observed after death. In cases of in-hospital deaths, early postmortem changes are typically observed because of the relatively short time between death and CT scanning. Decomposition (autolysis and gas production) is a late postmortem change, but it can occur during the early postmortem period. Understanding these changes and their mechanisms can aid in interpreting postmortem CT images and determining the cause of death (Table 1).

**Table 1:** Findings likely to be observed on CT in the early postmortem period of in-hospital deaths

Organs/Structures	Findings on CT in the early postmortem period of in-hospital deaths						
	Normal postmortem changes					Post-cardiopulmonary resuscitation changes	
	Livor mortis (= Postmortem hypostasis)	Rigor mortis	Changes secondary to cessation of circulation (due to hydrostatic pressure, membrane permeability, and blood clotting)	Decomposition		Artificial ventilation	Chest compression
				Gas production	Autolysis		
Head	Increased density of the venous sinuses		Brain swelling Obscuration of gray-white matter differentiation Increased density of the cerebrospinal fluid Blood clot of cerebral artery and superficial cerebral vein	Intravascular gas			Intravascular gas
Heart and great vessels	Hyper-density in the cavity of the gravity side	Thickening and hyper-density of the aortic and ventricular wall	Dilatation of the right heart system Aortic short diameter reduction and ovalization or flattening Cast-like blood clot in the pulmonary artery and aorta	Intravascular gas			Gas in the lumen of the heart and great vessels
Chest (other than heart and great vessels)	Ground glass density in the lung on the gravity side		Endotracheal and endobronchial fluid Pleural effusion				Rib fracture
Abdomen pelvis		Thickening and hyper-density of the aortic wall	Aortic diameter reduction and ovalization or flattening Mesenteric edema Increased density of the ascites after antemortem contrast-enhanced CT	Intraorganic and intracvascular gas, gas in the wall and dilatation of the gastrointestinal tract	Especially in the adrenal glands and pancreas Gastric perforation	Dilatation of the gastrointestinal tract Portal and mesenteric venous gas through the ischemic intestinal wall of the dilated intestinal tract	Intraorganic and intracvascular gas
Soft tissue	Skin thickening and subcutaneous edema	Hyper-density of the skeletal (striated) muscle		Intravascular gas			

The changes over time listed above are not always observed in all cases

### Livor mortis/postmortem hypostasis

**Fig. 1:**
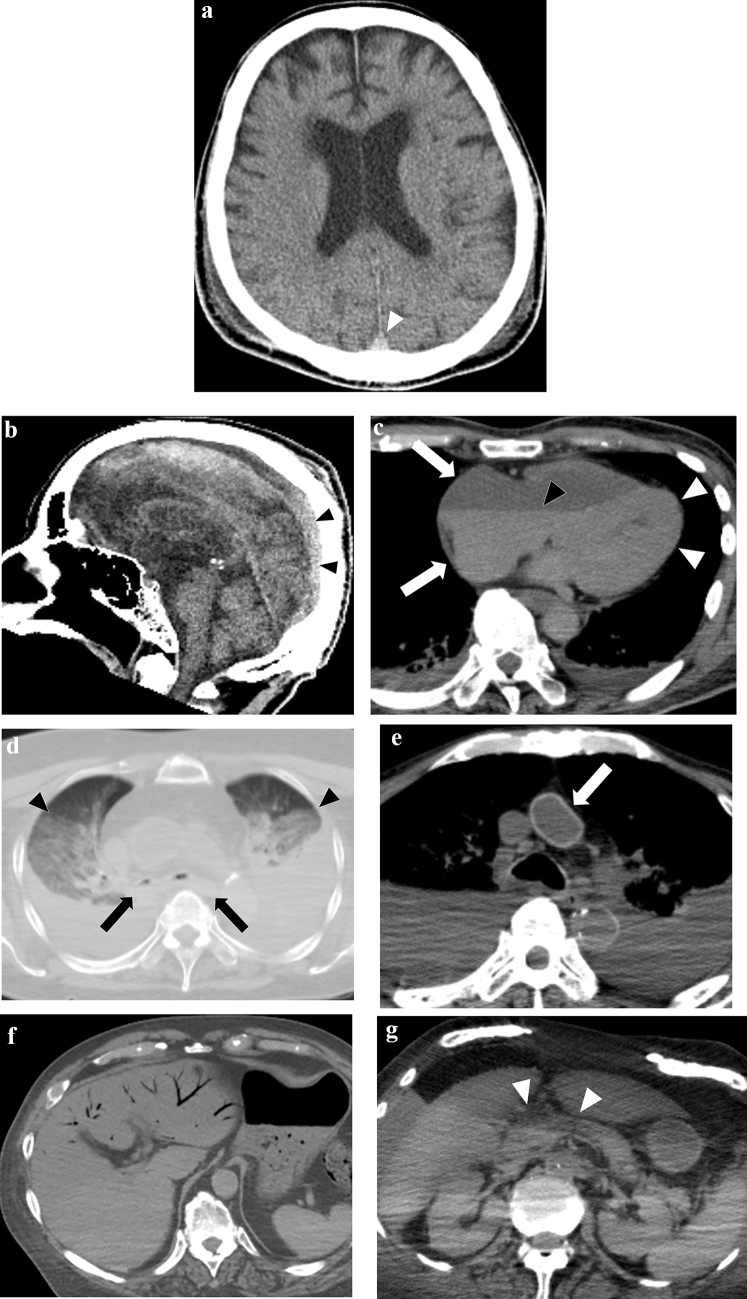
Representative CT images of early regular postmortem changes. **a**, **b** increased density in the superior sagittal sinus (arrowheads), brain edema (dorsally dominant), and loss of the differentiation between the gray and white matter. **c** thickened left ventricular wall (white arrowheads), hyperattenuating in the gravity side (black arrowhead), and dilatation in the right heart cavities (arrows). **d** fluid in the bilateral main bronchus (arrows) and the increased attenuation, such as ground-glass opacities with the horizontal plane formation on the gravity-dependent side of the lungs (arrowheads). **e** narrowed short diameter of the aorta and the hyperattenuating and thickened aortic wall (arrow). **f** intrahepatic portal venous gas (8 h elapsed from death without cardiopulmonary resuscitation). **g** obscuring of outline and swelling in the pancreas (arrowheads)

**Fig. 2:**
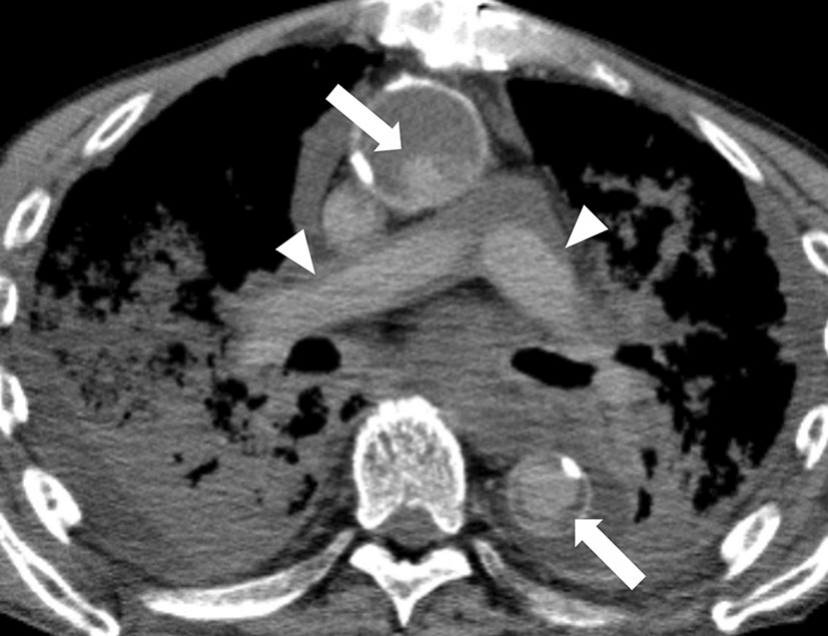
A cast-like hyperattenuating area in the pulmonary artery (arrowheads) and thoracic aorta (arrows) reflected the agonal stage or the long terminal phase from chronic disease

Livor mortis, also known as postmortem hypostasis, is a postmortem change that occurs as a result of circulatory arrest, which leads to a deprivation of blood supply. On CT images, this can manifest as hyper-attenuation in the dorsal venous sinuses, such as the superior sagittal and transverse venous sinuses (Fig. 1a and 1b) [18], and in the lumens of the heart and aorta, especially in the thoracic aorta (sometimes also in the inferior vena cava). This is due to the concentration of the hemoglobin molecule in sedimented erythrocytes and can sometimes lead to the formation of a fluid–fluid level (Fig. 1c) [19]. In the lungs, increased attenuation can be seen on the gravity side, resembling ground glass opacity with horizontal plane formation (Fig. 1d) [20], and this finding becomes more conspicuous over time [21]. Additionally, a distinct blood deposition with a horizontal plane in the lumens of the heart and aorta can indicate a hyper-fibrinolytic system due to acute death. In contrast, a cast-like hyperattenuating area can be seen in the pulmonary artery and aorta during the long death phase and in the agonal stage of chronic disease (Fig. 2) [22]. Subcutaneous edema and skin thickening can also occur and are generally symmetrical [23].

### Rigor mortis

Rigor mortis is a postmortem change that occurs as a result of muscle stiffness caused by chemical changes in the muscles after death. On CT images, this can manifest as an increase in the heart's ventricular wall thickness compared with antemortem images (Fig. 1c) [24], narrowing of short arterial diameters, and hyper-attenuation and thickening of the arterial wall, reflecting smooth muscle contraction. These findings are most prominent in large arteries, such as the aorta and pulmonary artery (Fig. 1e) [25–27]. Skeletal muscle, which is striated muscle, also exhibits increased attenuation on postmortem CT than on antemortem CT [28].

### Changes secondary to the cessation of circulation

After circulatory arrest, the vasculature is at a mean circulatory filling pressure, which is hydrostatic pressure (approximately 7 mmHg). This pressure is slightly higher than the antemortem right heart system diastolic pressure, which leads to a shift of blood volume to the right heart system after death and can result in dilatation of the right heart system and superior vena cava (Fig. 1c) [29] and narrowing of the aorta (Fig. 1e) [30, 31].

Additionally, there are other characteristic postmortem CT findings of brain parenchyma associated with cessation of circulation, such as edematous changes that increase the water content of the gray matter and obscure the distinction between gray and white matter (Fig. 1a) [32], narrowing of sulci and ventricles, and brain swelling when vasogenic edema is prominent (Fig. 1a) [33].

Loss of air in the trachea can also occur as fluid exudates from the lung interstitium into the alveoli and airways (Fig. 1d). This is a common finding when pleural effusion, lung consolidation, and atelectasis are present. Membrane hyperpermeability is thought to be one of the mechanisms [34].

The CT value of cerebrospinal fluid is higher in postmortem images than in antemortem images, but the degree of change can vary depending on the cause of death, antemortem pathology, and environmental conditions. In addition, there is a positive correlation between the CT values of the cerebrospinal fluid and elapsed time after death [35, 36].

There are few reports of postmortem CT findings for pleural effusion and ascites; however, the rate in pleural space fluid increased at 30 h, and the volume continued to increase until 40 h after death [37]. In addition, an elevated level of ascites attenuation on postmortem CT within 24 h after death relative to antemortem CT (AMCT) was confirmed in individuals who underwent enhanced AMCT shortly before death [38].

### Gas production

Gas production can occur in blood vessels and organs (Fig. 1f) because of the decomposition or presence of gas-producing bacteria in the vasculature and intestinal tract, even in cases where cardiopulmonary resuscitation was not performed [39]. (Gas production reflecting post-cardiopulmonary resuscitation changes is discussed later.) Gas is typically more prevalent 24 h after death but can also be seen in the early postmortem period, and its presence does not correlate with the time elapsed since death [40].

### Autolysis

Autolysis is a phenomenon in which tissues are digested by their enzymes after death, resulting in obscuring of organ outlines and changes in volume. In the early postmortem period, autolysis of the pancreas, spleen, and adrenal gland can occur (Fig. 1g) [41]. In the stomach, a condition called gastromalacia can occur, which can also cause gastric perforation because of the gastric juice. This postmortem change may be suspected on CT images when abdominal free air is observed despite the absence of other postmortem changes, such as intravascular or intra-organic gas [42], and must be distinguished from antemortem gastric perforation. Additionally, after 24 h postmortem, the brain shows a decrease in gray matter density due to autolysis [41].

## Cardiopulmonary resuscitation-related postmortem CT findings

Postmortem CT images may also show findings reflecting post-cardiopulmonary resuscitation changes performed before death. The effects of cardiopulmonary resuscitation before death are reflected in the postmortem CT findings (Table 1).

### Artificial ventilation

**Fig. 3:**
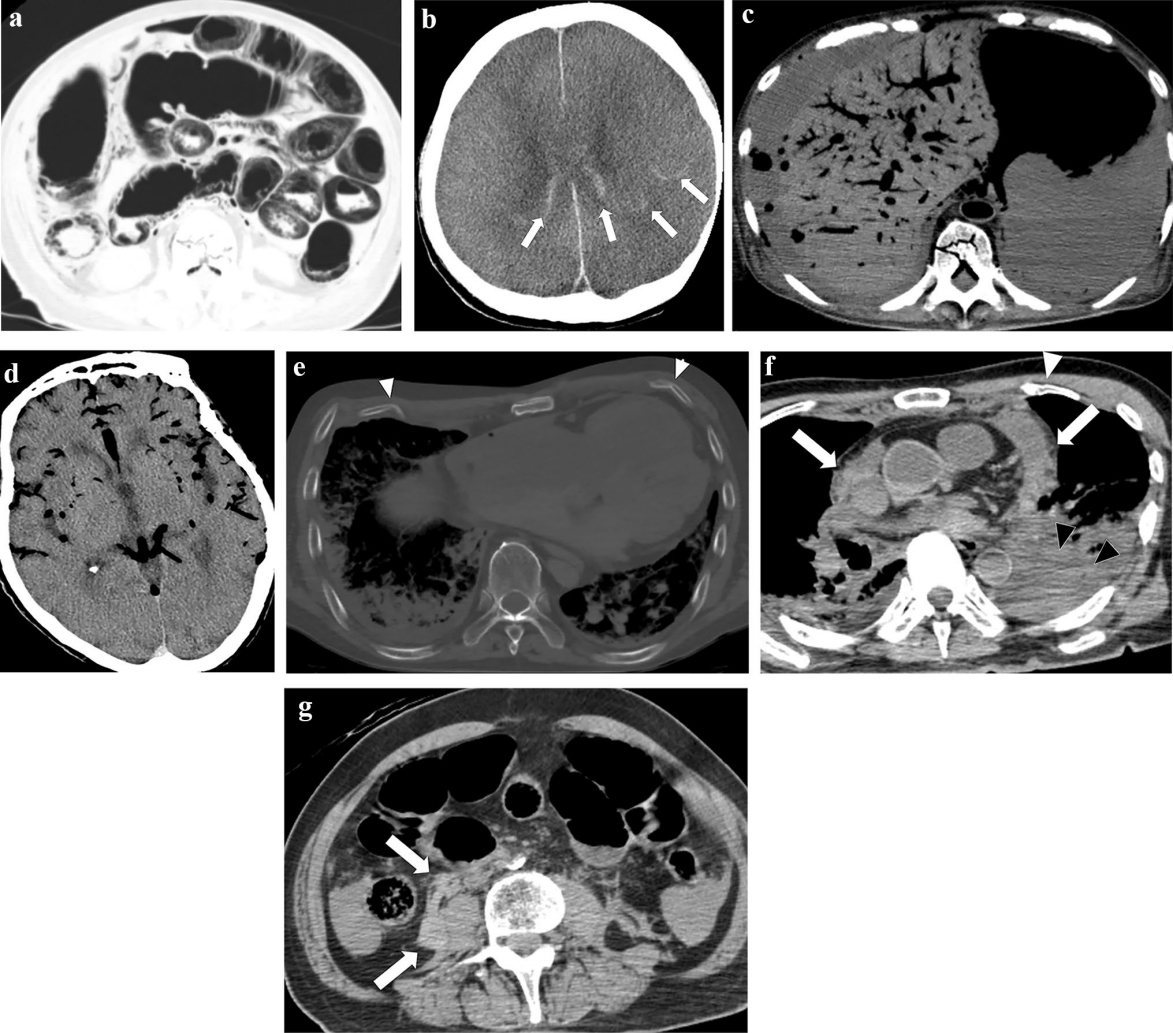
Representative CT images reflecting cardiopulmonary resuscitation. **a** extensive dilation of the gastrointestinal tract, gas in the intestinal wall and mesenteric vasculature. **b** the dilated medullary veins (arrows) near the lateral ventricles. **c** intraorganic and intravascular (broadly observed in the liver, abdominal aorta, vertebral body, and spinal canal) gas (scanned 10 h after death and also observed abdominal free air due to possible autolysis (unidentified bowel perforation by autopsy)). **d** gas in the arteries and veins in the brain (same case as Fig. 3c). **e** rib fractures in the anterior thoracic regions (arrowheads). **f** hemopericardium (arrows) due to cardiac rupture and sequel left hemothorax (black arrowheads) (also observed left rib fractures in the anterior thoracic regions (white arrowhead)). **g** right retroperitoneal hemorrhage (arrows) due to unknown cause confirmed by autopsy (same case as Fig. 3f)

Manual ventilation with a bag–valve–mask or similar device supplies air to the trachea, lungs, and gastrointestinal tract, resulting in extensive dilation of the gastrointestinal tract, primarily the stomach and small intestine (Fig. 3a). If the intestinal mucosa becomes ischemic owing to the cessation of circulation, gas from the lumen of the gastrointestinal tract can easily enter the intestinal wall (Fig. 3a). If the pressure on the ischemic intestinal wall is increased by the dilation and gas in the intestinal tract, intestinal gasses can pass through the ischemic wall, through the mesentery (Fig. 3a), and into the portal gas [43].

### Chest compression

Elevated white matter CT values cause the loss of gray–white matter differentiation in sudden death cases in which cardiopulmonary resuscitation was performed before death [44]. It is believed that the dilated medullary veins in the hypoxic brain under cardiac arrest become more congested by chest compressions, increasing white matter density (Fig. 3b) [45]. However, elevated white matter CT values can be seen even without cardiopulmonary resuscitation [46, 47].

Intravascular and intra-organic gas is frequently observed after chest compressions. The causes of this observation include the vaporization of dissolved gasses in the blood, air entry through the infusion route, and the formation of broncho-vascular fistulas (pressure trauma to the bronchi and pulmonary vessels) in combination with artificial ventilation [48, 49]. Chest compressions can spread intravascular gasses in the arteries in a prograde fashion and the veins in a retrograde fashion. The longer the duration of cardiopulmonary resuscitation, the more frequently gas is seen in multiple organs (Fig. 3c and d) [40].

Chest compression can easily cause rib fractures in the anterior and lateral thoracic regions (Fig. 3e) [50], and posterior rib fractures are usually considered inconsistent with chest compression. Chest compression can also complicate small pneumothorax, hemothorax, lung contusion, hemopericardium, and cardiac rupture (Fig. 3f) [51–53]. Intrapericardial and mediastinal hematomas may also occur. Owing to chest compression, sternal fractures can sometimes occur, and subcutaneous pre-sternal hematoma is sometimes observed following the use of a mechanical chest compression device [54]. Using mechanical chest compression devices can also result in unusual fractures of the mid-thoracic spine [55]. Abdominal hemorrhages, including peri-hepatic, peri-splenic, and retroperitoneal, are potential complications from chest compression attempts (Fig. 3g) [54, 56]. These findings can be explained when there is no other reason for antemortem pathology.

## Interpretation of postmortem CT

When interpreting postmortem CT images, it is important to consider normal postmortem changes and post-cardiopulmonary resuscitation changes that have already been discussed. The use of a checklist can aid in this process [57]. To interpret the images accurately, it is important to gather information such as the date and time of death, the date and time of CT imaging, medical history, and clinical course, and whether or not resuscitation was performed (if so, the details of the resuscitation). If a sequential autopsy is planned after the CT, it can be helpful to inform the autopsy physician of any relevant findings, even if they are negative. In summary, while the cause of death can sometimes be determined from postmortem CT images, it is important to recognize that the pathologist plays a central role in determining the cause of death, and the CT should be used as an adjunct [58].

## Diagnosable or non-diagnosable pathology on non-contrast-enhanced postmortem CT in in-hospital deaths

### Head and neck

**Fig. 4:**
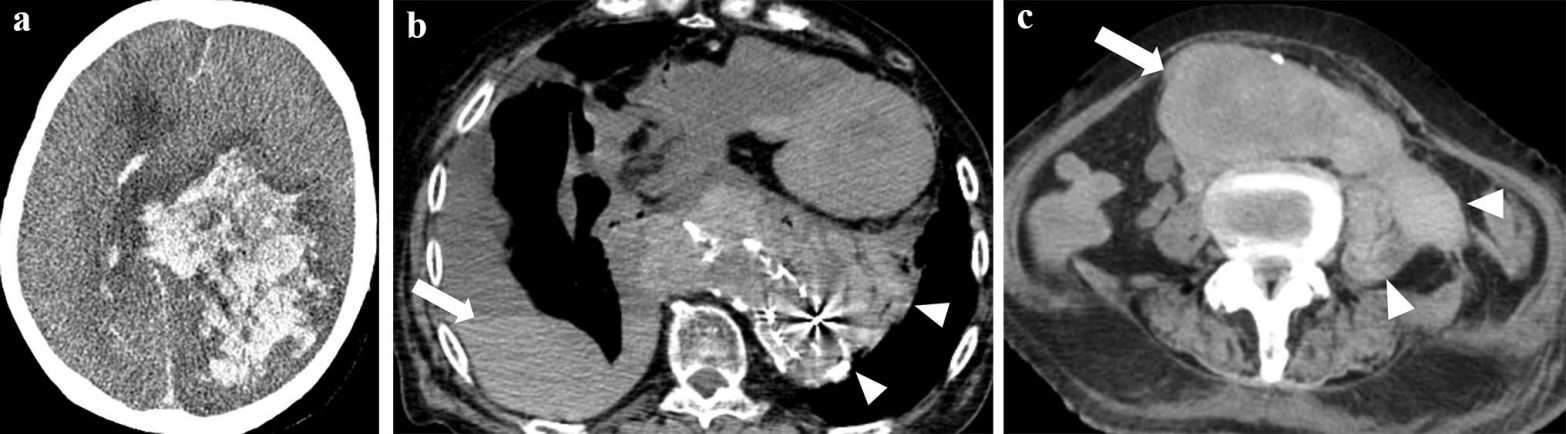
Examples of diagnosable fatal pathology on postmortem CT. **a** cerebral hemorrhage in the left parietal lobe with subfalcine herniation. **b** right hemothorax (arrow) due to ruptured thoracic aortic aneurysm after endovascular aortic repair (arrowheads). **c** ruptured abdominal aortic aneurysm (arrow) and massive retroperitoneal hemorrhage (arrowheads)

Gross intracranial hemorrhage and cerebral herniation, which are lethal intracranial lesions, can almost certainly be detected on postmortem CT images (Fig. 4a) [59, 60]. However, cessation of circulation reduces the amount of subdural hemorrhage in adults. This can be observed as a reduction in the volume of the subdural hematoma and midline deviation and an increase in hematoma CT value on postmortem CT images, compared with antemortem CT images [61, 62].

Additionally, significant diffuse cerebral swelling and obscuration of gray-white matter differentiation at a very early postmortem stage may indicate antemortem hypoxic-ischemic encephalopathy [63, 64]. These findings can sometimes be observed immediately after death and may be difficult to distinguish from normal postmortem changes. However, the brain in the very early postmortem period is in a state similar to hyperacute cerebral infarction. Cellular edema causes the swelling of individual brain cells and the narrowing of the spaces between cells, making it difficult for the overall brain volume to change significantly [65]. In the very early postmortem period, loss of gray–white matter differentiation because of cellular edema is rarely accompanied by severe brain swelling [32]. Changes in basal ganglia density may help distinguish these from postmortem changes [66]. In addition, a gross neck mass that narrows the airway can be determined by postmortem CT [67].

When performing diagnostic imaging on neonates and fetuses at the time of death, it is important to be aware that their skulls, and other organs and structures, such as the abdominal organs and eyeballs, are susceptible to severe deformation because of their lack of fixation. This can occur as a result of being placed on a CT cradle or suturing, leading to inaccuracies in the imaging results. Therefore, care should be taken to minimize mechanical deformation and ensure accurate imaging in these cases. [68, 69].

### Chest

Evaluating diffusely elevated pulmonary concentrations on postmortem CT can be challenging because of the high occurrence of postmortem pulmonary edema and hypostasis, which can obscure antemortem pathologies such as inflammation or tumors [12]. In children with established respiration, the air in the lungs may not be visible on postmortem CT images, making it difficult to diagnose conditions such as pneumonia before death [70]. However, certain conditions, such as ruptured thoracic aortic aneurysms, thoracic aortic dissection, pleural effusion, pneumothorax, and pneumo-mediastinum, can be diagnosed with high sensitivity on postmortem CT (Fig. 4b) [71]. Cardiac tamponade can also be diagnosed [72, 73]. Iatrogenic hematomas in the pericardium may occur owing to chest compression and must be distinguished from antemortem conditions [74]. Pulmonary arterial thrombo-embolization can be difficult to evaluate [33], but fat emboli in the pulmonary artery can be identified [74].

### Abdomen and pelvis

The detection of ruptured abdominal aortic aneurysms, intra-abdominal hemorrhage, gross abdominal masses, and gastrointestinal perforations, can be facilitated through postmortem CT imaging (Fig. 4c). However, the evaluation of gastrointestinal contents and walls can be challenging [75]. Postmortem CT images can be difficult to determine gastrointestinal hemorrhagic changes.

### Bones and soft tissues

**Fig. 5:**
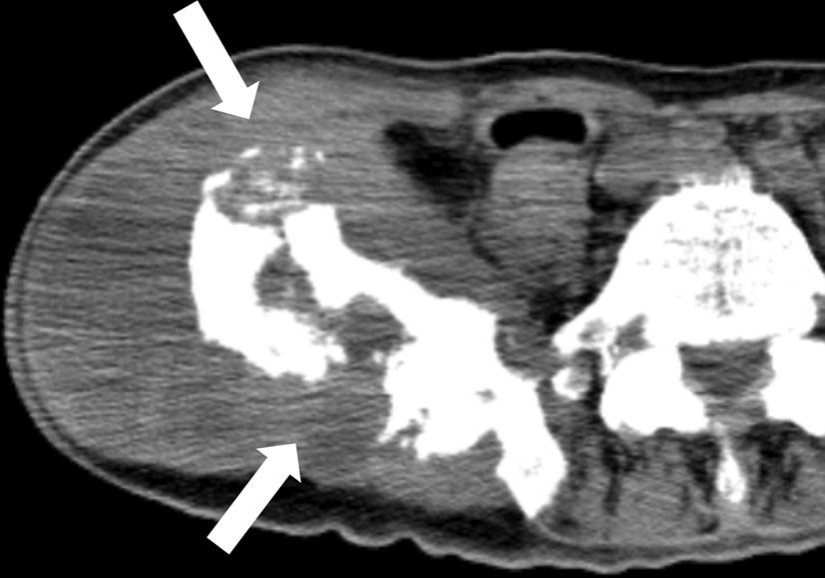
The superiority of postmortem CT for bony evaluation. Easy to understand right iliac metastasis (arrows) of lung cancer before sequential autopsy

Postmortem CT imaging can be used to elucidate bony lesions, such as the presence or absence of fractures and osteoplastic or osteolytic changes (Fig. 5) [23]. Additionally, postmortem CT imaging can reveal subcutaneous edema, large peripheral lacerations, and soft tissue hematomas [76]. These findings can aid in determining the cause of death and identifying any potential injuries and pathologies that occurred before death.

## Benefits of postmortem CT for pathologic autopsy in in-hospital death

Postmortem CT can provide detailed images of the body in its current state, allowing for a comprehensive evaluation of the cause of death [77]. The advantages of postmortem CT include: 1. The ability to determine the need for an autopsy by identifying key findings before autopsy. 2. The ability to plan autopsies by capturing images of the patient's condition before autopsy, reducing the risk of infection and exposure to hazardous materials for pathologists. 3. The ability to identify findings that may not be easily detected through traditional dissection methods, such as bony lesions. In some cases, postmortem CT may be sufficient to omit skeletal dissection during the subsequent autopsy [23].

Furthermore, postmortem CT allows for the preservation of the “as is” state of the body, which cannot be achieved once an autopsy has been performed. Additionally, postmortem CT images can be stored semi-permanently and reviewed again, providing superior objectivity. Furthermore, the reproducibility of the images can be helpful in discussions during investigations of medical accidents. Various devices and tubes should not be removed. They should remain in place for postmortem imaging, especially when medical malpractice is suspected or following resuscitation attempts [78].

## Diagnostic accuracy of non-contrast-enhanced postmortem CT to estimate the cause of death in cases of in-hospital deaths

Postmortem CT can aid in diagnosing the cause of death and major diagnoses related to death, surpassing clinical diagnosis alone in cases of in-hospital deaths (Table 2) [79–85]. The correlation rate of the immediate cause of death determined by postmortem CT and hospital autopsy is approximately 60‒70% [79, 82]. Similarly, the sensitivity for the cause of death is approximately 60‒70% with postmortem CT [79, 80, 84]. When combined with clinical information, postmortem CT can accurately diagnose respiratory failure [82]. On the contrary, in a large prospective cohort study validating postmortem images in diagnosing medicolegal deaths in adults, postmortem CT was more accurate than magnetic resonance imaging (MRI) in providing the cause of death, and postmortem CT could be acceptable for medicolegal purposes. However, the most common errors in identifying the cause of death by CT or MRI images based on conventional autopsy were ischemic heart disease, pulmonary embolism, pneumonia, and intra-abdominal lesions. Some common causes of sudden death are often omitted on postmortem CT and MRI and these imaging techniques cannot replace conventional autopsy [86].

**Table 2:** Potential of non-contrast-enhanced postmortem CT for cause-of-death and major diagnoses estimation in in-hospital death

First author	Year of publication	Number of reviewed autopsied cases	Age range (years)	Cause of death		Major diagnoses	
				Agreement (95%CI)	Sensitivity (95%CI)	Agreement (95%CI)	Sensitivity (95%CI)
Takahashi et al. [79]	2012	16	0–101	62.5% (35.4–84.8)	57.1% (28.9–82.3)	n/r	n/r
Westphal et al. [80]	2012	24	0–91	n/r	70.8% (48.9–87.4)	52.3% (36.7–67.5)	53.5% (37.7–68.8)
Wichmann et al. [81]	2012	47	Mean 63	n/r	n/r	n/r	71.4% (41.9–91.6)
Inai et al. [82]	2016	50	1–94	74% (n/r)	n/r	n/r	n/r
Arthurs et al. [83]	2016	82	0–15	n/r	n/r	32.73% (21.81- 45.90)	n/r
Sonnemans et al. [84]	2018	86	47–74	n/r	64% (53–75)	n/r	n/r
Ishida et al. [85]	2020	22	0–2	n/r	n/r	36% (n/r)	n/r

*n/r* not reported

When focusing specifically on in-hospital deaths in children, the detection rate of the main pathology related to the immediate cause of death using unenhanced postmortem CT in combination with clinical evaluation is approximately 30% [83, 85]. In cases of non-traumatic in-hospital deaths in children aged < 3 years, postmortem CT had a higher diagnostic sensitivity for respiratory system-related causes of death compared with clinical evaluation alone. However, regarding the causes of death related to cardiac and multi-organ systems, the diagnostic sensitivity of postmortem CT was significantly lower than that of clinical evaluation [85]. In children, an autopsy alone is not the best method for estimating the cause of death, and it is considered that autopsies should be used in combination with postmortem images. Postmortem CT is indispensable, especially for determining death due to trauma. On the other hand, in traumatic death cases of children aged 0 to 12 years, an autopsy alone is not the most reliable method for estimating the cause of death, and it is considered that autopsies should be used in combination with postmortem CT. Particularly, postmortem CT is superior to autopsy for detecting bone lesions in children [87].

## Conclusions

Postmortem CT plays a crucial role in postmortem investigations, particularly in cases of in-hospital death. It allows the accurate diagnosis of the cause of death and major diagnoses for specific cases, surpassing clinical diagnosis alone. Therefore, clinical radiologists are expected to have a role in the interpretation of postmortem CT images and the determination of the cause of death. This presents an opportunity and need for radiologists to expand their knowledge and experience in postmortem imaging and to contribute toward the understanding of death causes.
